# New kids on the CPET: age-appropriate outdoor cardiopulmonary exercise testing in preschoolers

**DOI:** 10.1007/s00421-021-04853-8

**Published:** 2022-01-16

**Authors:** Kathrin Rottermann, Annika Weigelt, Tim Stäbler, Benedikt Ehrlich, Sven Dittrich, Isabelle Schöffl

**Affiliations:** 1 Department of Pediatric Cardiology, Friedrich-Alexander-University Erlangen-Nürnberg, University Hospital Erlangen, Loschgestraße 15, 91054 Erlangen, Germany; 2 School of Clinical and Applied Sciences, Leeds Beckett University, Great Britain, Germany; 3 Department of Orthopaedic and Trauma Surgery, Section for Sports Medicine and Sport Orthopaedics, Klinikum, Bamberg, Germany

**Keywords:** Cardiopulmonary exercise test, CPET, Pediatric, Preschooler, Outdoor, Age-appropriate

## Abstract

**Purpose:**

Cardiopulmonary exercise testing (CPET) in preschoolers (4–6 years) represents a challenge. Most studies investigating CPET have been limited to older children (> 8 year). However, knowledge of the performance of small children is essential for evaluating their cardiorespiratory fitness. This study strives to compare a modified Bruce protocol with a new age-appropriate incremental CPET during natural movement running outdoors, using a mobile device.

**Methods:**

A group of 22 4–6-year-old healthy children was tested indoor on a treadmill (TM) using the modified Bruce protocol. The results were compared with a self-paced incremental running test, using a mobile CPET device in an outdoor park. The speeds were described as (1) slow walking, (2) slow running, (3) regular running, and (4) running with full speed as long as possible.

**Results:**

Mean exercise time outdoors (6,57 min) was significantly shorter than on the treadmill (11,20 min), $\dot{V}{O}_{2peak}$ (51.1 ml/min/kg vs. 40.1 ml/min/kg), RER (1.1 vs. 0.98) and important CPET parameters such as $\dot{V}E$_*max*_, O_2_pulse, heart rate and breath rate were significantly higher outdoors. The submaximal parameter OUES was comparable between both the tests.

**Conclusions:**

Testing very young children with a mobile device is a new alternative to treadmill testing. With a significantly shorter test duration, significantly higher values for almost all cardiopulmonary variables can be achieved without losing the ability to determine VT1 and VT2. It avoids common treadmill problems and allows for individualized exercise testing. The aim is to standardize exercise times with individual protocols instead of standardizing protocols with individual exercise times, allowing for better comparability.

## Introduction

Lack of physical activity and the often-related problem of obesity are well-known risk factors for cardiovascular diseases (Boyer et al. 2015; Werneck et al. 2019). Among the health-related physical fitness components in children and adolescents, we know cardiorespiratory fitness (CRF) to be the most important marker (Ortega et al. 2007; Ruiz et al. 2009). Higher levels of CRF during early childhood are favorably associated with multiple health indicators (Carson et al. 2017). They especially result in lower cardiovascular disease risk factors during later life (Nordström, Högström, and Nordström 2014) and reduce the risk of becoming overweight across puberty (Ortega et al. 2007).

Cardiopulmonary exercise testing (CPET) is a commonly performed, non-invasive method to evaluate cardiac symptoms and assess functional capacity in children (Paridon et al. 2006) and can be considered as safe (Ghosh et al. 2015). In very young children (4–6 years) though, CPET is highly challenging. Bicycle testing is usually not feasible in this age group due to, cognitive, anatomic and technical limitations (average height for a 6-year-old is about 118 cm, cutoff for bicycle testing according to the German medical products law is 120 cm) (Neuhauser 2013). Therefore, exercise testing can only be performed on a treadmill (TM). Requirements for a representative treadmill test are good motor skills and a high motivation, both difficult to achieve in this age group. Most studies investigating CPET have, therefore, been limited to older children (> 8 years of age) (Ghosh et al. 2015; Duff et al. 2017; Leger et al. 1988; Akkerman et al. 2010; Armstrong and Welsman 2001; Armstrong et al. 1991; van der Steeg and Takken 2021; Blanchard et al. 2018; Bongers et al. 2016).

Since children have relatively underdeveloped knee extensors, treadmill testing is generally preferred over cycle ergometry (CE) in young children (Bar-Or 2004). The most common TM protocol is the Bruce protocol which was originally designed for diagnosing coronary artery disease in middle-aged men (Bruce et al. 1963). It has since become the most common TM protocol, even in children from the age of 4 years on (Wessel et al. 2001). In this protocol, speed and inclination are increased every 3 min (Bruce et al. 1963). However, increasing the incline during a treadmill test represents a high demand for those relatively underdeveloped knee extensors and may lead to premature local leg fatigue, causing the children to stop running before achieving maximal performance (Duff et al. 2017). In addition, the rather large increases between the different steps and a step-duration of 3 min each are difficult, as boredom and demotivation step in (Duff et al. 2017). Several attempts have been made, to modify the Bruce protocol for a better suitability for children (van der Cammen-van Zijp et al. 2009; Dubowy et al. 2008; Eiberg et al. 2005; Tuan et al. 2019, 2018).

Furthermore, field-based tests for the indirect estimation of $\dot{V}O$_2peak_ have been proposed, and inter alia, derived as a consequence of lacking equipment, trained personnel or even financial aspects (Mora-Gonzalez et al. 2017; Leger et al. 1988). One approach is the adapted 20 m Shuttle-run test for preschool children, the so-called 20mSRT-PREFIT (Mora-Gonzalez et al. 2017). However, in this approach, a validation of data is not possible without true cardiopulmonary exercise data (Tomkinson et al. 2019).

The development of mobile cardiopulmonary exercise testing instead offers a whole new opportunity for accurate measurements of $\dot{V}{O}_{2peak}$ in children (Schoffl et al. 2020a). In a recently published study examining cardiopulmonary field testing using a mobile CPET device, young school children were successfully tested by running in an outdoor park. The children were able to increase the speed according to their own capabilities, allowing for age- and fitness-adapted testing and keeping the test duration comparable (Schoffl et al. 2020a). One limitation of this first study was the fact that it compared the field test with indoor CE testing. Therefore, in this study CE testing was used for the indoor protocol. As maximal exertion during biking and running cannot directly be compared, no conclusion could be drawn as to the benefits of outdoor vs. treadmill testing. Still, this was the first study to investigate field testing in children (7–10 years of age).

In this study, we want to implement the upper mentioned field test (Schoffl et al. 2020a) in preschool children (4–6 years) and compare it to the ramped modified Bruce protocol on the treadmill which was developed by Dubowy et al. (2008) and is actually recommended by the German Association for Pediatric Cardiology (DGPK) for TM testing in children. Thus, the comparison between the indoor and outdoor tests was more realistic as walking and running were used in both the tests.

## Materials and methods

The study was approved by the Ethics Committee of the University of Erlangen-Nuremberg (159_19B).

### Subjects

Twenty-two healthy children between 4 and 6 years of age legally agreed to participate in this study. Written informed consent was obtained from each child and the respective parent, using age-appropriate consent forms. All participants were Caucasian, non-obese, and healthy. None was taking medications. Anthropometry data were obtained using a stadiometer and electronic scale (Seca 704 S, Hamburg, Germany).

### Pre-test questionnaire

Cardiorespiratory fitness is highly associated with physical activity (Grgic et al. 2018). Classification of the specific physical fitness of each subject according to sports participation and kinder garden transport habits (walking, cycling, bus) was established using a questionnaire proposed by van der Cammen-van Ziip et al. (2009), which has already been used in a previous study with older children (Schoffl et al. 2020a). According to the results from the questionnaire, the children were then classified into low (only sports education classes), moderate (physical education classes and participation in organized sports up to 2 h per week) or high (gymnastic lessons and participation in organized sports more than 2 h per week) with regards to their physical activity (Schoffl et al. 2020a).

### Cardiopulmonary exercise testing

Measuring of gas-exchange was performed via a continuous breath-by-breath method and averaged over intervals of 15 s. We used a mobile equipment with a small, low-dead-space respiratory valve (88 ml) and a pediatric mouthpiece (Metamax^®^, Cortex Biophysic GmBh Co., Leipzig, Germany), which was fitted on the children’s back. The cardiopulmonary exercise equipment weighed 600 g. The backpack for carrying the Metamax was adjusted as to fit the small proportions of preschool children.

According to the recommendations of Wasserman et al., ventilatory thresholds VT_1_ (first ventilatory threshold, start of anaerobiosis and accumulation of lactate) and VT_2_ (second ventilatory threshold, point of ventilatory compensation of lactic acidosis) were determined using the V-slope method (Beaver et al. 1986). Single regression analysis was used to determine OUES slope by plotting $\dot{V}{O}_{2}$ (ml/min) against the logarithm of $\dot{V}E$ (ml/min), reflecting the relation between oxygen uptake $\dot{V}{O}_{2}$ (ml/min) and minute ventilation $\dot{V}E$ (ml/min) during incremental exercise, thus showing the effectiveness of $\dot{V}{O}_{2}$ (Akkerman et al. 2010; Baba et al. 1996).

Physiological criteria for completion of a valid peak $\dot{V}{O}_{2 peak}$ test included two of the following three criteria: (1) peak HR within 5% of the age-predicted maximum, (2) RER ≥ 1.0, and (3) volitional fatigue. Criteria for $\dot{V}{O}_{2 max}$ were (1) peak HR ≥ 200 beats per minute (bpm), (2) HR ≥ 85% of the age-predicted maximum, (3) RER ≥ 1,1 (Tuan et al. 2018) or (4) leveling off, respectively, monitored both indoors and outdoors. Since the latter is difficult to determine in children, special consideration was applied when observing a true plateau (Armstrong et al. 1991). The subjects were asked, not to consume food or drinks rich in carbohydrates 2 h prior to the tests. Emphasis was given on similar test conditions including time of day or temperature. Due to COVID-19 pandemic contact restrictions, this could not be guaranteed for all the outdoor tests, as some had to be postponed and then be performed at a later date. Still, extreme temperature conditions were avoided. Indoor tests were performed in an air-conditioned room (20 °C) within the hospital. Outdoor temperatures varied from around 5 °C to 25 °C. Indoor tests always took place prior to the outdoor tests as a 12-lead ECG could only be used during the indoor test and we wanted to exclude arrhythmias in a secure environment.

### Treadmill exercise testing

Indoor cardiorespiratory exercise testing was performed on a common treadmill (Cosmed^®^) with safety precautions such as supervision by trained staff, proper shoes and protection with rope and harness. Continuous monitoring of HR took place via 3-lead ECG (Custo^®^). To familiarize the preschoolers with the TM and the gas measuring equipment, the children were allowed to examine the mask and TM characteristics were explained thoroughly. Every child used a short warm-up phase to get familiarized with the TM. All subjects were tested according to the ramped modified Bruce protocol, developed by Dubowy et al. (2008) and recommended by the DGPK (Deutsche Gesellschaft für pädiatrische Kardiologie). This protocol starts at 2.5 km/h, speeding up every 90 s by 0.5 km/h. TM-inclination starts at a speed of 3 km/h by 3%. Every 90 s, inclination is increased by 3%, up to a maximum of 21%. Speed continues to increase by 0.5 km/h at every step. When maximum inclination is reached (21%), only speed is increased further by 0.5 km/h every 90 s. Volitional fatigue, cardiac arrhythmia or repeated statement of unwillingness to continue in spite of verbal encouragement were reasons for terminating the test.

### Outdoor test

To guarantee a sufficient recovery, there was a minimum of 1 week in between indoor and outdoor tests. We attempted to keep time in between both tests as short as possible, to keep anthropometry data and fitness level comparable at the time of the second test.

A short warm-up phase comparable to the one conducted during the TM protocol was used in the outdoor setting. Prior to the test, each child was fitted with a heart rate (HR) monitor (Polar H7 Bluetooth Smart 4.0^®^ heart rate sensor, Kempele, Finland) and the same mobile cardiopulmonary exercise equipment (Metamax^®^, Cortex, Leipzig, Germany) used indoors. The test consisted of a self-combined with a researcher-paced incremental test with a total of four velocity steps on a flat ground in an outdoor park, previously tested in older children (Schoffl et al. 2020a). Instructions were given to each child before the test. The first step consisted of relaxed walking for 2 min, then speeding up to an easy jog for another 2 min. The third increment was explained as a faster speed, which children would be able to maintain for 2 min. The last step meant running as fast for as long as possible. This step was compared to trying to catch a friend running at a faster pace. To control the children’s speed and the time of each step, every child was accompanied by an experienced researcher. The main objective here was to slow down the children for the first two steps if necessary and encourage them during the last two steps, and of course, to ensure the safety of each participant. Speed was adapted to each child and its individual fitness level. A GPS tracker was used for measuring speed during the test. The tracker was mounted on the backpack and carried by the child. It is extremely small and weighs no more than 10 g. The maximum duration of each stage was set at 2 min. Thus, the distance covered by each child differed according to their own speed during each stage. All tests were undertaken by the same researchers. The children were instructed to use adequate footwear and clothing. An experienced pediatrician, trained in emergency treatment, was on site for the whole time of the test.

### Statistical analysis

SPSS for Windows 12.0^®^ (SPSS Inc., Chicago, IL) was used for all statistical analyses. All measured values are reported as means and standard deviations, and categorical data are presented as absolute numbers. Levine’s *F* test was used for scanning on homogeneity of variance. Normal distribution was verified using the Kolmogorov–Smirnov test. For normally distributed variables, paired *T* tests were applied. All tests were 2-tailed. Data were considered statistically significant with a *p* value of less than 0.05. Artwork was designed using SPSS for Windows 12.0® and Power Point for Windows (Office 2019).

## Results

### Subjects

Overall, 22 children participated in the study. We were able to test 21 children on the TM, and 19 children successfully performed the field test. Three children could not be tested outdoors, as the interval between the tests was too long as a consequence of the Corona related lock-down. One child could not be persuaded to run on the TM but was extremely motivated to run outdoors. Out of the 22 children, 9 were boys and 13 were girls. Table 1 represents their age, anthropometric data, as well as their sports participation and their kinder garden transport habits. The children recruited in this study proved to be rather physically active. Only 32% of the subjects used the bus to get to the kinder garden/school, 68% walked or went by bike. Most of the children participated in extracurricular sport activities (68%), 41% were classified as high with regard to physical activity, 27% were classified as moderately active and 32% as low.

**Table 1:** Anthropometric data as well as sports participation and kinder garden transport habits as means and standard deviation

	Girls (*n* = 13)	Boys (*n* = 9)
Age (years)	5.1 (± 0.9)	6.0 (± 0.7)
Height (cm)	113.5 (± 7.0)	116.3 (± 7.5)
Weight (kg)	18.5 (± 3.5)	20.8 (± 2.1)
BMI (kg/m^2^)	14.3 (± 1.9)	15.4 (± 1.5)
Sports participation	30.8% low 30.8% moderate 38.5% high	33.3% low 22.2% moderate 44.4% high
Kinder garden transport habits	30.8% bus 46.2% foot 23.1% bike	33.3% bus 22.2% foot 44.4% bike

### Comparison of the outdoor test with the treadmill

Both tests were well tolerated by the children except by one girl, who was too frightened of the TM, to perform indoor. Comparing indoor to outdoor, the children reported enjoying the outdoor test more and were noticeably more motivated to run outdoors. None of the tests had to be stopped due to an accident, pathological arrhythmias or other adverse events. While most children stopped the TM test due to unwillingness to keep going or local fatigue in the legs, the outdoor tests were stopped when the children could not maintain their speed during the last step.

The parameters from the two cardiopulmonary exercise tests are represented in Table 2. Most of the CPET values showed a significant difference between the two tests, with higher values for the outdoor than the treadmill test: $\dot{V}{O}_{2peak}$, peak velocity, RER, $\dot{V}E$_*max*_, O_2_pulse, maximum heart rate (HR), and maximum breath rate (BR). The submaximal parameter OUES, a parameter of cardiopulmonary functional reserve, was comparable between both the tests.

**Table 2:** Means, standard deviations and exact *p* values of the cardiopulmonary exercise test parameters of the indoor versus the outdoor test (* depicts a significant difference between the two tests)

Parameters	Indoor test	Outdoor test	*p* values
$\dot{V}{O}_{2}$ at VT1 (ml/kg/min)	24.8 (± 7.2)	26.9 (± 4.3)	0,170
$\dot{V}{O}_{2}$ at VT2 (ml/kg/min)	37 (± 5.6)	46.1 (± 8.8)	0,061
Peak heart rate (bpm)*	177.2 (± 17.3)	186.9 (± 9.7)	0,037
Heart rate at VT1 (bpm)	140.2 (± 16.8)	129.4 (± 12.2)	0,055
Heart rate at VT2 (bpm)	177 (± 17.6)	177 (± 13.4)	1,000
Peak O2-pulse (ml/heart beat)*	4.4 (± 1.1)	5.2 (± 1.1)	0,003
Peak $\dot{V}E$ (ml/min)*	29.6 (± 8.7)	37.9 (± 8.4)	0,000
Peak breath rate (br/min)*	59.7 (± 10.1)	66.7 (± 6.1)	0,016
OUES (oxygen uptake efficiency slope)	1.0 (± 0.2)	1.1 (± 0.2)	0,271
$\dot{V}E/\dot{V}{CO}_{2}$*	34.2 (± 6.2)	25.9 (± 2.0)	0,000
Peak velocity (km/h)*	5.4 (± 0.8)	8.3 (± 1.8)	0,000

**Fig. 1:**
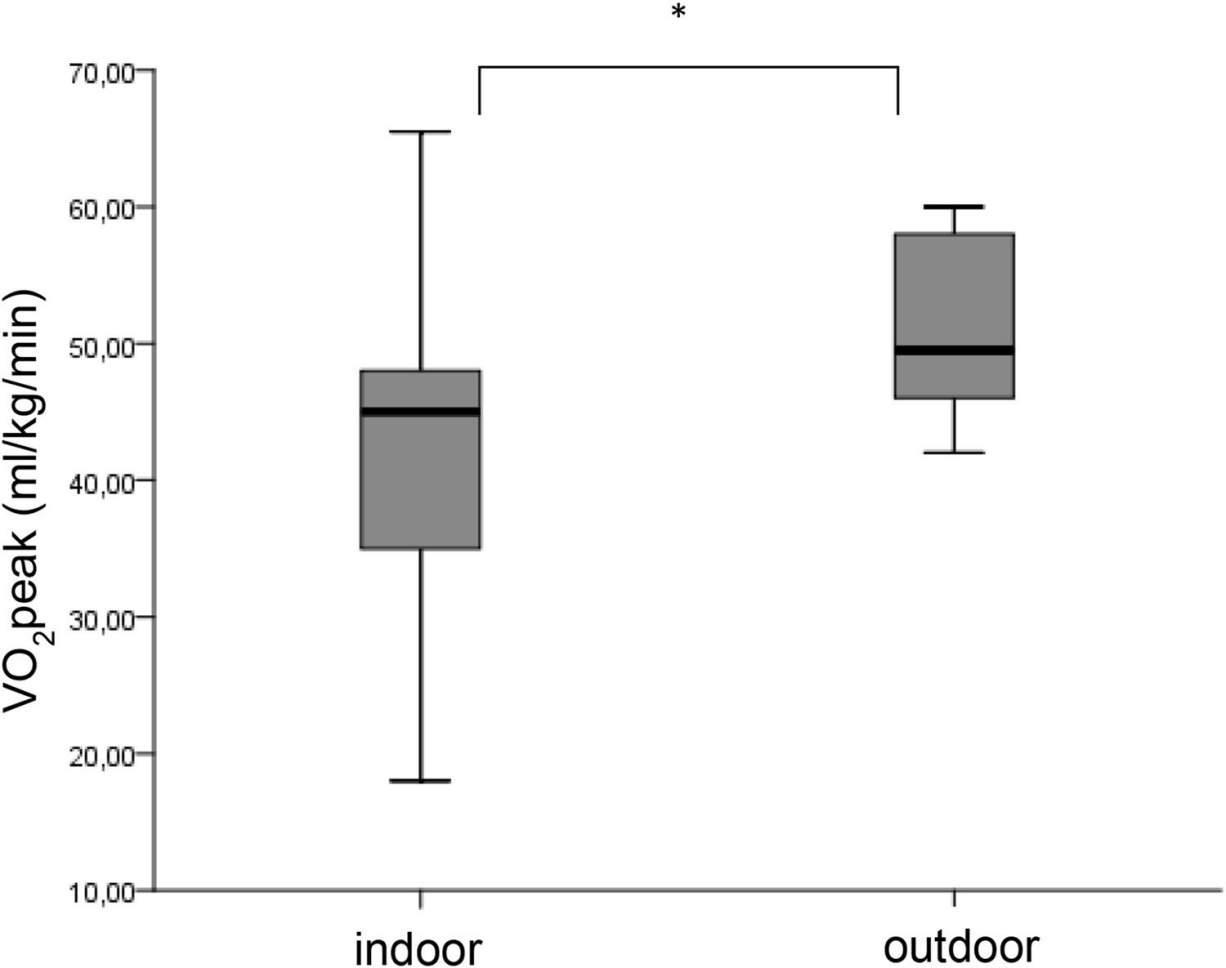
$\dot{{\varvec{V}}}{O}_{2peak}$ during the indoor and the outdoor tests (* represents a significant difference between the two tests)

**Fig. 2:**
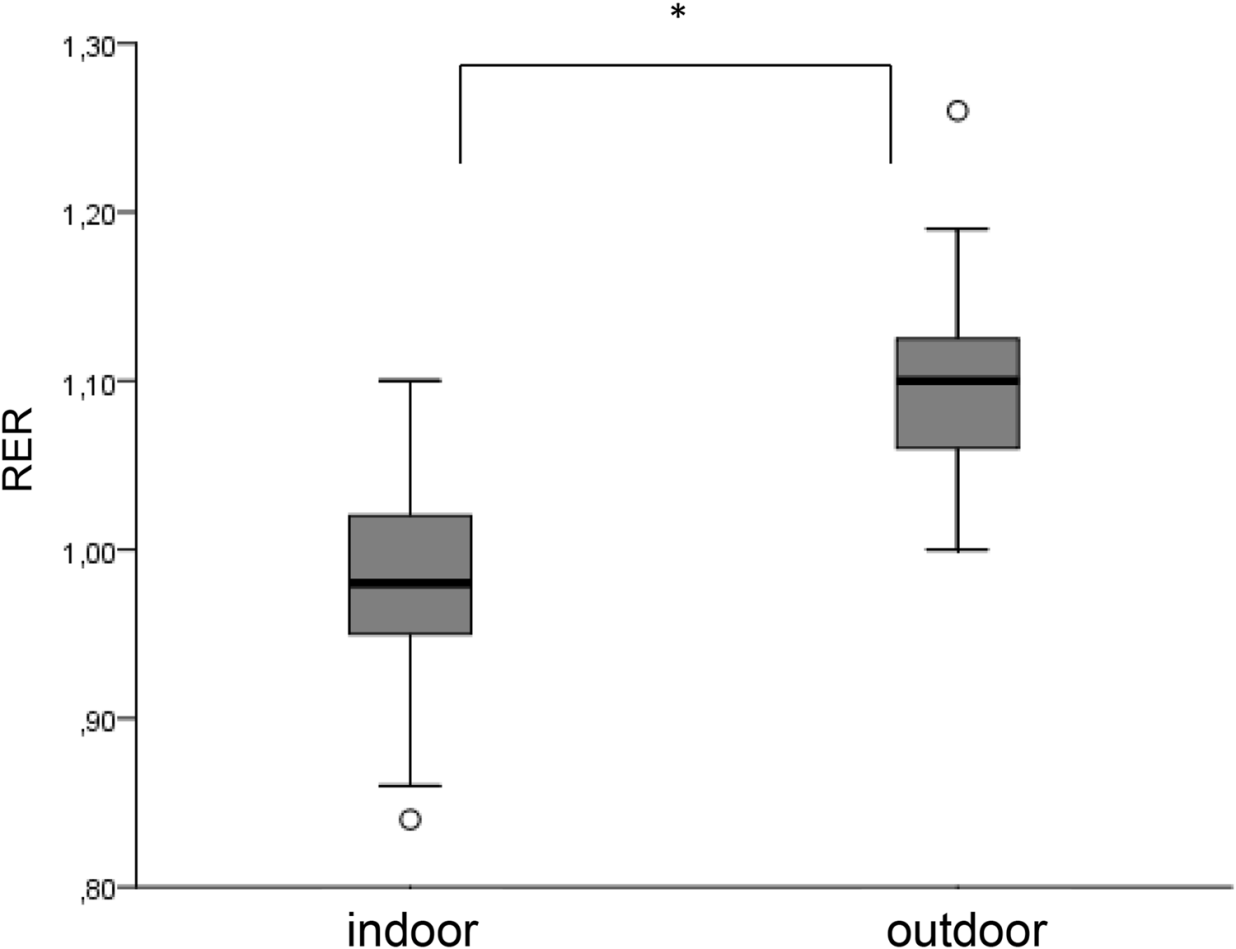
RER during the indoor and the outdoor tests (* represents a significant difference between the two tests)

**Fig. 3:**
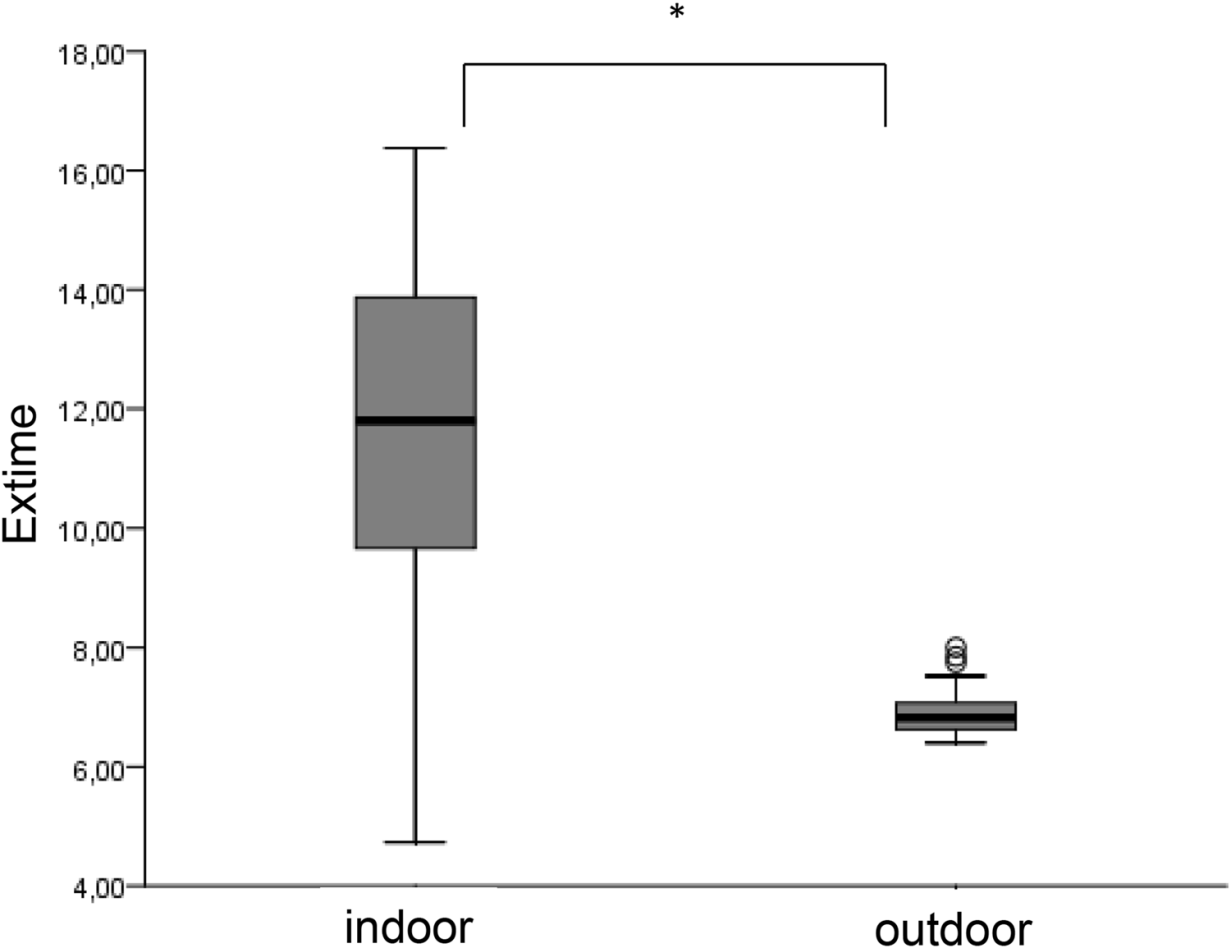
Duration of exercise time during the indoor and the outdoor tests (* represents a significant difference between the two tests)

We observed a significantly higher $\dot{V}{O}_{2}peak$ outdoors than indoors (*p* value 0.000) with a mean value of 51.5 ml/min/kg (± 6.6) vs. 40.6 ml/min/kg (± 9.2) (Fig. 1). During the outdoor test, all children reached the second ventilatory threshold (VT2) and a realistic $\dot{V}{O}_{2}peak$, whereas during the indoor test only 6 children (29%) reached VT2. Four children (19%) reached a plateau in $\dot{V}{O}_{2}peak$ during the outdoor test, whereas none of the children reached this criterion of maximum cardiopulmonary exercise during the treadmill test. All children achieved a respiratory exchange ratio (RER) ≥ 1.0 during the outdoor test, but only 33% (7 children) during the indoor test. Average RER during the field test was 1.10 (± 0.1), which was significantly higher (*p* value 0.000) than during the treadmill test with a mean RER of 0.97 (± 0.1) (Fig. 2). Mean exercise time outdoors was significantly shorter (6 min 57 s, ± 30 s) than indoors (11 min 20 s ± 3 min), *p* value 0.000 (Fig. 3). $\dot{V}E$_*max*_ indoors was significantly lower (*p* value 0.000) with 29.6 ml/min (± 8.7) versus 37.9 ml/min (± 8.4) outdoors, O_2_pulse indoors was 4.4 ml/heart beat (± 1.1) compared to outdoors 5.2 ml/heart beat (± 1.1), *p* value 0.003, maximum heart rate (HR) differed significantly (*p* value 0.037) from indoors 177.2 bpm (± 17.3) to outdoors 186.9 bpm (± 9.7) and maximum breath rate (BR) as well showed a significant difference (*p* value 0.016) from indoors 59.7 br/min (± 10.1) to outdoors 66.7 br/min (± 6.1) (Table 3).

**Table 3:** Anthropometric data as well as sports participation and kinder garden transport habits as means and standard deviation

	Girls (*n* = 13)	Boys (*n* = 9)
Age (years)	5.1 (± 0.9)	6.0 (± 0.7)
Height (cm)	113.5 (± 7.0)	116.3 (± 7.5)
Weight (kg)	18.5 (± 3.5)	20.8 (± 2.1)
BMI (kg/m^2^)	14.3 (± 1.9)	15.4 (± 1.5)
Sports participation	30.8% low 30.8% moderate 38.5% high	33.3% low 22.2% moderate 44.4% high
Kinder garden transport habits	30.8% bus 46.2% foot 23.1% bike	33.3% bus 22.2% foot 44.4% bike

### Speeds recorded during the outdoor tests

 Table 4 shows the mean velocity achieved by the children at each step during the outdoor test. The children`s average speed was 3.16 km/h (± 0.44 km/h) for the first step, 4.29 km/h (± 0.96 km/h) for the second step, 5.65 km/h (± 1.25 km/h) for the third step and 7.22 km/h (± 1.45 km/h) for the last step. Maximum speed (v_max_) outdoors was significantly higher than indoors (8.31 ± 1.8 km/h vs. 5.38 ± 0.8 km/h), but with increasing incline (Table 2).

**Table 4:** Average speeds during each step in the outdoor test as means and standard deviation

vmean step 1	vmean step 2	vmean step 3	vmean step 4	vmax
3.16 ± 0.44 km/h	4.29 ± 0.96 km/h	5.65 ± 1.25 km/h	7.22 ± 1.45 km/h	8.31 ± 1.8 km/h

## Discussion

Evaluation of the cardiorespiratory fitness of healthy children and those who suffer from a chronic cardiopulmonary disease is essential for determining cardiovascular risks but also for potential disease management. Due to anatomic limitations, CPET with very young children can only be performed on the TM. But this is often problematic, as the required motor skills may not yet be sufficiently developed in this age group (Hebestreit 2004). 

Our study population consisted of a total number of 22 children aged 4–6 years, which is comparable to other studies (Schoffl et al. 2020a). Average height (114 cm) was below the minimum height required for bicycle testing (Hebestreit 2004). Both indoor and outdoor testing represented no health risk to the children. The preschoolers in this study showed a clear preference for the outdoor test. They were more reluctant to run on the treadmill, presenting coordinative difficulties, culminating in one indoor-dropout due to fear of the TM. This observation has previously been described (van der Cammen-van Zijp et al. 2009) but was not evident during the outdoor testing, where the children were highly motivated to run.

Hebestreit et al. (2004) reported children to prematurely quit during the first few seconds of a new step, likewise implying an increment of workload (Hebestreit 2004). The preschoolers in this study showed comparable behavior on the TM. The reason for this phenomenon is probably linked to the motor-skill-related problems occurring on the TM as they were especially noticeable during low speeds and at the start of a new step. Another reason for the premature termination of tests at the beginning of a new step could be the increasing inclination of the TM leading to leg fatigue due to underdeveloped knee extensors in this age group (van der Cammen-van Zijp et al. 2009). Furthermore, uphill running requires higher energy expenditure than running on a flat ground (Cavagna et al. 1964). To address this specific problem when testing children, efforts have been made to develop new TM protocols for children without inclination of the treadmill (Duff et al. 2017). Duff et al. 2017 used a protocol (BCCH) where the incline stayed at a constant 1% starting at a speed of 2.0 mph, increasing by 0.5 mph every minute until volitional fatigue. In comparison, the Bruce protocol simultaneously increases the speed and grade of the treadmill every 3 min. As a result, the BCCH-protocol requires children to run at an earlier stage and at faster speeds. Furthermore, it addresses the problem of local leg fatigue, which is often a complaint in the Bruce protocol. Endpoint data such as $\dot{V}$E, $\dot{V}$O_2_, $\dot{V}$CO_2_, RER and HR were comparable between both protocols. Nevertheless, the BCCH-protocol requires more exercise time than the Bruce protocol and was only tested in children aged 10–18 years, which is one of the reasons why we did not use it for our preschoolers. In our clinical experience, long exercise time leads to boredom and premature quitting of the test, especially in younger children. On top, the German Association of Pediatric Cardiologists (DGPK) recommends the TM protocol by Dubowy, which is the one we used indoors, and it is, therefore, probably the most common TM protocol used for testing children in Germany.

Both protocols performed by Eiberg and LeMura, as well as the Bruce protocol, used an inclination on the TM, which we did not choose due to the problem of local leg fatigue while running uphill and the upper mentioned fact of the problems encountered using protocol by Dubowy.

We commonly observed that the children were able to chatter a few seconds after TM test termination, whereas after the outdoor tests, none of the children was able to hold up to a normal conversation due to exertion. Studies about the talk test as a marker of exercise training intensity suggest there is a strong correlation between a decrease in spoken words and training above the ventilatory threshold (Reed and Pipe 2014). We presume that running on the TM did not lead to the same level of cardiorespiratory exertion as running outdoors.

When working with children, motivation and fun are crucial factors. Hence, it is important to note that all the children enjoyed the outdoor test more than running on a treadmill. This observation has previously been described in a study comparing outdoor testing with indoor cycle testing (Schoffl et al. 2020a).

Essentially, the children were able to reach significantly higher RER values running outdoors than during the TM test (RER outdoor 1.10 vs. indoor 0.97). This parameter is representative of the maximum exertion achieved by the test person. A study on 5–6-year-old children has defined maximum effort as RER values > 1.0 (LeMura et al. 2001). Average values of 1.05–1.10 have been reported for testing 6–7-year-old-children on a treadmill (Eiberg et al. 2005). In a TM-study on preschoolers using a ramp protocol instead of a stepwise increase of the workload, RER values as high as 1.03–1.18 were achieved (Tuan et al. 2019). Our results for the outdoor test are comparable, showing maximum cardiorespiratory exertion. However, the mean RER values achieved on the TM using the modified Bruce protocol were below the recommended value of 1.0 (LeMura et al. 2001), suggesting that cardiorespiratory exertion was not achieved in our cohort. Therefore, this protocol may not be suitable for testing preschoolers. The same outdoor protocol has previously been used in 7–10-year-old children (Schoffl et al. 2020a). The RER values achieved in this study were even higher (RER 1.19 in boys and 1.25 in girls), reflecting on the fact, that testing older children is more feasible (Schoffl et al. 2020a).

In our study, most of the CPET values showed a significant difference between the two tests, with higher values achieved during the outdoor test. The peak oxygen consumption $\dot{V}{O}_{2}peak$, which is a surrogate parameter of cardiopulmonary fitness, was much higher outdoors than on the TM (51.5 ml/min/kg vs. 40.6 ml/min/kg). This, we think, is due to a better cardiopulmonary exertion, reflected by the higher RER (Schoffl et al. 2020a). Other studies determining $\dot{V}{O}_{2}peak$ in 5–7-year-old children on the TM and on a stationary bike, recorded comparable results with values ranging from 44.8–48.5 for $\dot{V}{O}_{2}peak$ (Eiberg et al. 2005; LeMura et al. 2001). The same outdoor test performed by older children (7–10-year-olds) also provided comparable results with mean $\dot{V}{O}_{2}peak$ ranging from 50.0 m/min/kg in girls to 52.8 ml/kg/min in boys (Schoffl et al. 2020a). The outdoor test, therefore, seems to provide reasonable results even in children as young as 4 years of age.

There has been a variety of studies in children, reporting about higher maximum HR when tested on the TM than on the CE (Armstrong et al. 1991; Boileau et al. 1977; Cumming 1985), including one examining preschoolers (LeMura et al. 2001). A possible explanation for this discrepancy is the limiting influence of underdeveloped knee extensor muscle in young children, inducing local muscle fatigue and premature termination of the CPET (van der Cammen-van Zijp et al. 2009). In our study, the HR was significantly higher outdoors compared to the indoor TM test (187/min vs. 177/min). Again, this could be due to the same phenomenon, since an increase in inclination will also lead to local muscle fatigue of the underdeveloped knee extensors and the TM test demanded going uphill in the protocol, whereas the outdoor protocol did not. The same outdoor test used in this study elicited mean heart rates of 192.5 in boys and 199.3 in girls, who were 7–10 years old (Schoffl et al. 2020a). The most reasonable explanation for this discrepancy is the difficulty to reach maximum exertion in children so young. Other TM-studies reported maximum heart rates ranging from 194 ± 11/min in 6-year-olds (van der Cammen-van Zijp et al. 2010), up to an average of nearly 200/min in 5–6-year-old children (LeMura et al. 2001). However, a study with a comparable age group (5.58 years in girls and 5.86 years in boys) recorded comparable values to our study (178–184/min) (Tuan et al. 2019). These considerations underline the fact that better cardiopulmonary exertion was reached during the outdoor test compared to the indoor TM test, which was also reflected by the significantly higher values of $\dot{V}E$_*max*_, O_2_pulse, and maximum breath rate (BR).

The oxygen uptake efficiency slope (OUES), an age- and sex-dependent parameter of submaximal cardiopulmonary effort (Bongers et al. 2016), provides a valid measure of cardiopulmonary fitness in children who cannot reach maximal exertion (Bongers et al. 2011). As OUES and $\dot{V}{O}_{2peak}$ strongly correlate (Bongers et al. 2016), the OUES can be used as a surrogate parameter for $\dot{V}{O}_{2peak}$, especially in study populations, where maximal CPET results are difficult to achieve due to, e.g., motivational issues (Ten Harkel and Takken 2019). Furthermore, $\dot{V}{O}_{2peak}$ and OUES show excellent interest reproducibility and reliable results among the same study population (Baba et al. 1999). This is consistent with our study results as the preschoolers show comparable results for OUES both during indoor TM and outdoor testing. Despite the comparable results for OUES, the children were able to achieve significantly higher maximal CPET values during the outdoor testing than on the TM. This characterizes the OUES as a test-independent, reproducible, submaximal parameter.

Average peak velocity was significantly higher during the outdoor tests (8.3 km/h vs. 5.4 km/h), which is a consequence of running without inclination outdoors but with increasing steepness on the TM. Compared to the mean peak velocity obtained in a comparable study on 7–10-year-olds (10.11 km/h), the preschoolers were significantly slower (Schoffl et al. 2020a). The mean speeds recorded at each step could be used for developing an age-appropriate TM test with age-appropriate speeds (first step: 3.16 km/h, second step: 4.29 km/h, third step: 5.65 km/h, fourth step: 7.22 km/h).

The fact that TM protocols working for adults might not fit for children has led to the development of more age-appropriate test protocols for children. Several authors have altered the Bruce protocol, by shortening the steps, decreasing the increments of speed and inclination (van der Cammen-van Zijp et al. 2009; Dubowy et al. 2008) and starting with a higher speed (Eiberg et al. 2005). This has led to a reduction in mean test time to values as low as 7.28 min (Eiberg et al. 2005). With these average test durations in mind, we used a test protocol with a defined maximum duration time of 8 min. Mean outdoor test duration in our study was 6.57 min., a time that was significantly shorter than the average test duration of 11.20 min. using the common TM protocol. Even though, this test duration seems comparably short, better cardiopulmonary exertion was achieved and ventilatory thresholds were easily discernible. We believe that keeping exercise times short will prevent boredom and consequently premature termination of CPET and that comparable exercise times are more important than comparable test protocols for a better comparability of CPET data (Schoffl et al. 2020a). As these children grow older, their relative speed may, therefore, increase for each step, however, the test duration will remain comparable.

The study limitations in our study are the same as in a previous study using mobile CPET equipment in the park (Schoffl et al. 2020a). Namely, the need for special equipment (mobile CPET and specialized ECG), the restrictions due to bad weather conditions and the need for trained test personnel for ensuring the proper test protocol as well as the safety of the child.

Concluding, common TM problems such as fear of TM testing, local leg fatigue, boredom and subsequent premature test ending were evaded by letting the children use natural movement patterns in a natural habitat, namely running on flat ground in an outdoor park (Duff et al. 2017). In our opinion, proper cardiorespiratory exertion does not depend on the applied protocol but on its feasibility in the corresponding study population. All the children in this study preferred the outdoor testing over running on the TM, and consistently showed significantly higher values of cardiorespiratory exertion. It is our aim to standardize exercise times, using individual protocols adapted to the capacity of each child, its stage of disease and chronological age and physiological development instead of standardizing protocols with individually lasting exercise times, thus allowing for a better long-term comparability.

## Abbreviations

BR: Breath rate
CE: Cycle ergometry
COVID-19: Corona virus disease 2019
CPET: Cardiopulmonary exercise test
CRF: Cardiorespiratory fitness
DGPK: Deutsche Gesellschaft für pädiatrische Kardiologie
ECG: Electrocardiogram
HR: Heart rate
O_2_pulse: Oxygen pulse
OUES: Oxygen uptake efficiency slope
RER: Respiratory exchange ratio
TM: Treadmill
$\dot{V}{E}_{max}$: maximal voluntary ventilation
v_max_: Maximum speed
$$\dot{V}{O}_{2}$$: Oxygen uptake
$\dot{V}{O}_{2peak}$: Peak oxygen uptake
$\dot{V}{O}_{2 max}$: Maximum oxygen uptake
VT1: First ventilatory threshold
VT2: Second ventilatory threshold
